# Retrotransposon insertion as a novel mutational event in Bardet‐Biedl syndrome

**DOI:** 10.1002/mgg3.521

**Published:** 2018-11-28

**Authors:** Erika Tavares, Chen Yu Tang, Anjali Vig, Shuning Li, Gail Billingsley, Wilson Sung, Ajoy Vincent, Bhooma Thiruvahindrapuram, Elise Héon

**Affiliations:** ^1^ Genetics and Genome Biology Hospital for Sick Children Toronto Ontario Canada; ^2^ Institute of Medical Science University of Toronto Toronto Ontario Canada; ^3^ The Centre for Applied Genomics Hospital for Sick Children Toronto Ontario Canada; ^4^ Ophthalmology and Vision Sciences Hospital for Sick Children Toronto Ontario Canada

**Keywords:** Bardet‐Biedl syndrome, BBS, *BBS1*, ciliopathy, human genome, mutation, repetitive element, SVA, transposable element

## Abstract

**Background:**

Bardet‐Biedl syndrome (BBS) is an autosomal recessive pleiotropic disorder of the primary cilia that leads to severe visual loss in the teenage years. Approximately 80% of BBS cases are explained by mutations in one of the 21 identified genes. Documented causative mutation types include missense, nonsense, copy number variation (CNV), frameshift deletions or insertions, and splicing variants.

**Methods:**

Whole genome sequencing was performed on a patient affected with BBS for whom no mutations were identified using clinically approved genetic testing of the known genes. Analysis of the WGS was done using internal protocols and publicly available algorithms. The phenotype was defined by retrospective chart review.

**Results:**

We document a female affected with BBS carrying the most common *BBS1* mutation (*BBS1*: Met390Arg) on the maternal allele and an insertion of a ~1.7‐kb retrotransposon in exon 13 on the paternal allele. This retrotransposon insertion was not automatically annotated by the standard variant calling protocols used. This novel variant was identified by visual inspection of the alignment file followed by specific genome analysis with an available algorithm for transposable elements.

**Conclusion:**

This report documents a novel mutation type associated with BBS and highlights the importance of systematically performing transposon detection analysis on WGS data of unsolved cases.

## 1 BRIEF REPORT

Bardet‐Biedl syndrome (BBS:MIM209900) is a complex disease characterized mainly by severe photoreceptor degeneration, truncal obesity, postaxial polydactyly, autism‐like behavior, cognitive impairment, hypogonadism, renal anomalies, among other secondary features (Gerth, Zawadzki, Werner, & Heon, 2008; Habibullah & Mohiuddin, 2009; Heon et al., 2005; Kerr, Bhan, & Heon, 2015; Weihbrecht et al., 2017). BBS is phenotypically and genetically heterogeneous, and demonstrates considerable overlap with other ciliopathies such as Joubert syndrome (JBST) (Beales, Elcioglu, Woolf, Parker, & Flinter, 1999; Billingsley et al., 2010; Branfield Day et al., 2015; Deveault et al., 2011; Gerth et al., 2008; Heon et al., 2016; Kerr et al., 2015). Biallelic mutations have been identified in at least 21 BBS genes (Heon et al., 2016; Khan et al., 2016), all of which are involved in primary cilia structure and/or function (Alvarez‐Satta, Castro‐Sanchez, & Valverde, 2017; Khan et al., 2016). Approximately 80% of the clinically examined BBS cases (Forsythe & Beales, 2015) have been associated with biallelic mutations in one of the 21 BBS genes, of which Bardet‐Biedl syndrome‐1 gene (*BBS1: *209,901) is the most frequently mutated (Billingsley, Deveault, & Heon, 2011). Currently, reported mutations in BBS are as follows: missense, nonsense, frameshift, and nonframeshift variants; copy number variation; alternative splicing; and a few descriptions of complex rearrangements (Billingsley et al., 2011; Khan et al., 2016; Weihbrecht et al., 2017). In this study, we report a novel exonic retrotransposon insertion into *BBS1*, which represents a new type of mutational event for BBS patients and may play a role in the missing heritability of this condition. This study was approved by the Research Ethic Board of the Hospital for Sick Children and met the Tenets of the Declaration of Helsinki.

**Figure 1:**
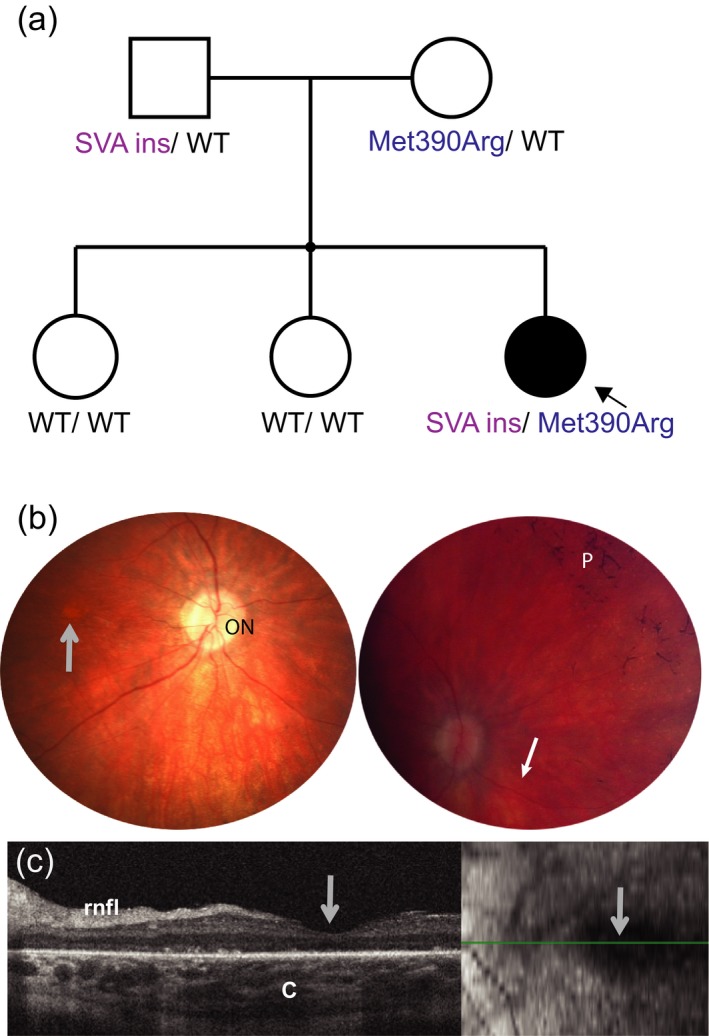
(a) Pedigree of the affected family and *BBS1* variant segregation; (b) retinal photography of the right eye centered on the optic nerve (ON, left image) and on the nasal retina (right image). Gray arrow locates the foveal area which shows atrophy (center of the retina), and a blunted reflex. Filled white arrow (right) points toward narrowed (very thin) vessels, p: bone spiculing pigmentary deposits typical of retinal degeneration. (c) Optical coherence tomography (OCT) of the right eye centered on the fovea (gray arrow) shows markedly disruption of outer retinal layers including the photoreceptor outer and inner segments, and the outer nuclear layer. rnfl: retinal nerve fiber layer, c: choroidal layer. Insert to the right shows the area scanned (green line). The quality of the images was limited due to the patient's ability to participate

The proband was a female from a sibship of three, born to nonconsanguineous parents of European origin (Figure 1a). She presented with night blindness, constriction of visual field, and photophobia at the age of nine (Table 1). Distance visual acuity was reduced (right: 20/400, left: 20/600), and severe generalized rod and cone photoreceptor degeneration was documented on an electroretinogram (ERG) (Supporting information Figure S1). This correlated with the observed retinal structural changes (macular atrophic changes and pigmentary retinal changes and vessel attenuation; Figure 1b,c). It was not possible to perform a visual field test due to the developmental delay. However, based on the small residual central island of photoreceptors documented by optical coherence tomography (OCT), it is likely that the fields were very constricted (Figure 1c). Other features included obesity (BMI 37.6 at 13 years old), postaxial polydactyly, fatty infiltration of the liver, elevated lipids, autism, and absence‐type seizures (Table 1). The clinical phenotype was consistent with BBS (Beales et al., 1999; Daniels et al., 2012) and part of the phenotype variability associated with *BBS1* mutations (Deveault et al., 2011).

**Table 1:** Phenotype summary of the proband

Onset symptoms	Age 9–10 years (nyctalopia, visual field constriction)
VA (14 years)	20/400 (right eye), 20/600 (left eye)
Anterior segment	Normal
Retinal exam (15 years)	Macular atrophy, vessel attenuation, bone spiculing changes
ERG amplitude (1 years)	Severe rod‐cone dystrophy
Neurological findings (age 13 years)	Absent Seizures, autism
	Normal Brain MRI
	Head circumference: 57 cm
Kidney (age 13 years)	Normal structure and function
Liver (age 13 years)	Fatty Infiltration, normal transaminases
Lipids^a^ (age 13 years)	Cholesterol 6.4 mmol/L (⇑)
	Triglyceride 8.63 mmol/L (⇑)
Heart	Situs Solitus, levocardia
Spleen	Mild splenomegaly
Digits	Postaxial polydactyly × 3 limbs
Weight	BMI 38.9
Menarche	13 years
Developmental	Delayed
Other	Recurrent ear infections, strabismus

a Normal lipid values; cholesterol: 0.65–2.5 mmol/L, triglyceride: 0.40–103 mmol/L.

Genetic testing initially done using a CLIA‐approved laboratory performing next‐generation sequencing on 19 BBS genes revealed a heterozygous *BBS1 *(NM_024649.4) variant: c.1169 T>G, p.Met390Arg, the most common mutation in *BBS1*. Segregation analysis of the variant by Sanger sequencing confirmed that the mutation was heterozygous in the proband and that the mother was a carrier. No other potentially pathogenic mutation was found in *BBS1* or other BBS‐associated genes. No significant copy number variation (CNV) was detected by microarray analysis.

**Figure 2:**
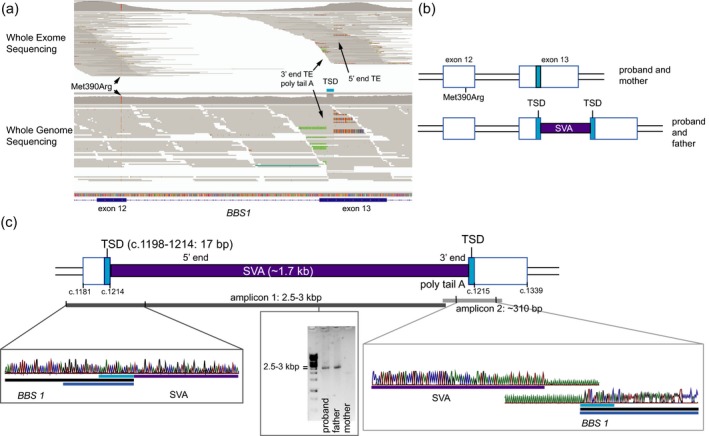
Disease‐causing mutations in the proband with retrotransposon insertion detail. Pathogenic mutations are represented in purple, Target Site Duplication (TSD) is shown in cyan, and exon is represented in blue. (a) Schematic representation of the retrotransposon insertion in exon 13 of *BBS1* in Integrative Genome Viewer using the whole exome and the whole genome sequence alignment maps, respectively (Robinson et al., 2011). Nucleotides matching the reference are gray; unmatched A, T, C, and G nucleotides to reference are color coded in green, red, blue, and orange, respectively. (b) Schematic diagram of the two alleles comprising both *BBS1* mutations (Met390Arg and the exonic TE insertion in exon 13) observed in the proband and parents. (c) Detailed characterization of the novel SVAF insertion detected in this study and schematic representation of the results used to assemble the mutation map. Each PCR product is represented as a bar overlapping the region they comprise in the mutation map. Chromatograms from Sanger sequencing represent the *BBS1*‐SVAF junction

Whole genome sequencing (WGS) was performed, and single‐nucleotide variants (SNVs), insertions/deletions (indels), and CNVs were annotated with our standard in‐house variant filtration pipeline approach (Supporting information Data S1 and Figure S2). The five genes with biallelic variants (three genes with homozygous variants and two genes with compound heterozygous variants) were excluded because these were either platform based errors (*ZFPM1*, *SLCA6*), inframe deletion in a polymorphic region (*UBXN11*), or the variants did not segregate with the disease phenotype (*DNAH8, IGSF3*) (Supporting information Figure S2). Since BBS best fits the proband's phenotype, a CNV analysis was performed for *BBS1 *(for which a heterozygous missense mutation was previously identified; Met390Arg)*,* and for two other ciliopathy‐related genes (for which heterozygous variants were also identified in the proband's WGS filtering (Supporting information Figure S2): *NPHP4* (NM_015102:exon26:c.3644+1G>C), *CEP78* (NM_032171:exon15:c.1801–1G>C)). CNV was not detected for any of the three genes (Supporting information Data S1, Figure S2), but during the visualization of the binary alignment map (BAM) for these three genes, we identified discrepant reads (split reads) in exon 13 of *BBS1 *only (Figure 2a). The split portion of the forward reads (127 bp) was 98.5% identical to a retrotransposon of the category SINE/VNTR/*Alu *subtype *F* (SVAF) in RepeatMasker (http://www.repeatmasker.org). This novel transposable element (TE) insertion on *BBS1* had not yet been reported in the database of retrotransposon insertion polymorphisms dbRIP (http://dbrip.brocku.ca/searchRIP.html). The corresponding mate pairs of the split reads were mapped at different parts of the human genome (hg19) where TEs of the similar category had been mapped. The TE insertion (*BBS1*:NM_02649.4:c.1214–1215ins (1700_1800);1198_1214) was validated using PCR and Sanger sequencing and verified to be paternally inherited (Figure 2b,c, Supporting information Data S1, Figure S3, Table S1). The final sequence (GenBank MH395756, Supporting information Data S2) had higher similarity (95%–97.7%) with multiple SVA types B, D, and mostly F from the human whole genome sequence (hg19 in UCSC—https://genome.ucsc.edu/‐ using RepeatMasker track). The allele carrying the SVA insertion was expressed in patient‐derived lymphoblast cell line; these results showed that BBS1 protein is translated up to AA residue 406, followed by insertion of 46 new AA residues (corresponding to the SVA) before reaching stop codon (Supporting information Figure S4).

The shorter PCR segment amplification (Supporting information Data S1, Figure S3) did not identify this particular SVA insertion in our cohort of 24 genetically unsolved BBS probands. Additionally, the insertion was not found in 2,504 control individuals from 1,000 genomes phase three databases, for which MELT, an automatic TE detection method was applied (Gardner et al., 2017; Rishishwar, Tellez Villa, & Jordan, 2015).

We validated our finding using Mobster (Thung et al., 2014), an algorithm designed to detect transposable element insertions from next‐generation sequencing data (Supporting information Data S1). We then applied this method to five of unsolved BBS probands for which WGS data were available. However, no potentially disease‐causing TEs were identified for them.

We could not automatically detect the SVA insertion from the whole exome sequencing (WES) of the proband using the same protocols applied to WGS, mostly due to the lower number of reads bearing the *BBS1* and SVA junction in WES. However, some of those split reads can be observed in the WES reads map (Figure 2a).

## 2 DISCUSSION

We report for the first time an SVA retrotransposon insertion in a *BBS1 *exon as a disease‐causing mechanism for BBS.

DNA transposable elements (TE) are mobile DNA elements that occupy nearly half of the human Genome (http://genome.ucsc.edu), yet are poorly explored (Kazazian & Moran, 2017). The aggregate length of these sequences is 40 times greater than protein‐coding exons (Kazazian & Moran, 2017; Lander et al., 2001; Mills, Bennett, Iskow, & Devine, 2007). Active TEs account for around 0.02% of the human genome and belong to the retrotransposon class (Kaer & Speek, 2013; Lander et al., 2001). Retrotransposons can be transcribed into RNA, reverse transcribed into cDNA, which is then reinserted in the genome at a new location (Kaer & Speek, 2013; Lander et al., 2001). It is estimated that one de novo insertion happens every 10–100 live births. The most abundant retrotransposon in the human genome is the Long Interspersed Nuclear Element (LINE; 17%), followed by Alu (11%) and composite SVA repeats (0.2%) (Kaer & Speek, 2013; Lander et al., 2001; Solyom & Kazazian, 2012).

The role of transposable elements in disease has only been minimally explored. However, these can be pathogenic by inserting themselves into coding or regulatory portions of genes, facilitating chromosomal rearrangements, duplications, and deletions, among other mechanisms (Mills et al., 2007). Only a few cases of human retinal degeneration have been reported to be caused by inserted retrotransposons; an *Alu* insertion on exon 9 of *MAK*, and intronic *Alu* insertion in *OPA1* (Bujakowska, White, Place, Consugar, & Comander, 2015; Gallus et al., 2010; Tucker et al., 2011). A few cases have also been reported in animals; an L1 insertion in rodent *Nr2e3* (Chen, Rattner, & Nathans, 2006), an intronic SINE insertion in canine *FAM161A* (Downs & Mellersh, 2014), and an intronic insertion in equine *TRPM1* (Bellone et al., 2013). Recently, two cases affected with nonBBS ciliopathy phenotypes were associated with retrotransposon insertions; a L1 in exon 7 of *CC2D2A *and an *Alu* in exon 16 of *ALMS1* (Takenouchi et al., 2017; Taschner et al., 2016). A recent study of BBS cases also found that 40% (6 out of 15) of the CNV deletions detected in their cohort (in *BBS1*, *BBS4*, *BBS5*, and *IFT74*) were mediated by *Alu*–*Alu* recombination (Lindstrand et al., 2016).

The high amount of active TE in the human genome, and the technical difficulties associated with their detection suggests that they are likely an underexplored category of disease‐causing mutational events. Therefore, the implementation of TE detection protocols in the standard pipeline for search of disease‐causing variants can significantly increase diagnostic yield and improve patient care. Traditional methods such as candidate gene sequence and microarray analysis are unsuitable to capture large insertions such as TEs. Large‐scale sequence analysis such as whole exome and whole genome sequencing allows identification of TEs but require specific algorithms for automatic detection (Ewing, 2015; Gardner et al., 2017; Thung et al., 2014), which are not systematically incorporated in the current practice (Takenouchi et al., 2017). The WES is less efficient to WGS in automatic TE detection because some of the genomic segments including the junction between the gene of interest and inserted TE are eliminated at the exome capture step (Tucker et al., 2011). Therefore, standard implementation of Mobster or other TE detection methods should be planned forward for WGS data. Currently, there is only one “population frequency database” of nonreference TEs insertions available, making it difficult to determine the prevalence of this mutational event in disease or in the general population (Gardner et al., 2017; Rishishwar et al., 2015). The incorporation of TE insertion frequencies from the 1,000 genomes, and other additional population databases, will allow for more efficient identification of potentially pathogenic TEs. Further larger‐scale implementation of such detection methods will allow for the discovery of more disease‐causing variants and provide a clearer idea of the role of TEs in the population and disease processes.

## CONFLICT OF INTEREST

The authors have no conflict of interest to declare.
