# Supplementary figures and images for: Retrotransposon insertion as a novel mutational event in Bardet‐Biedl syndrome
Source: Mol Genet Genomic Med. 2018 Nov 28;7(2):e00521. doi: 10.1002/mgg3.521 (PMC6393654; doi:10.1002/mgg3.521)

2016

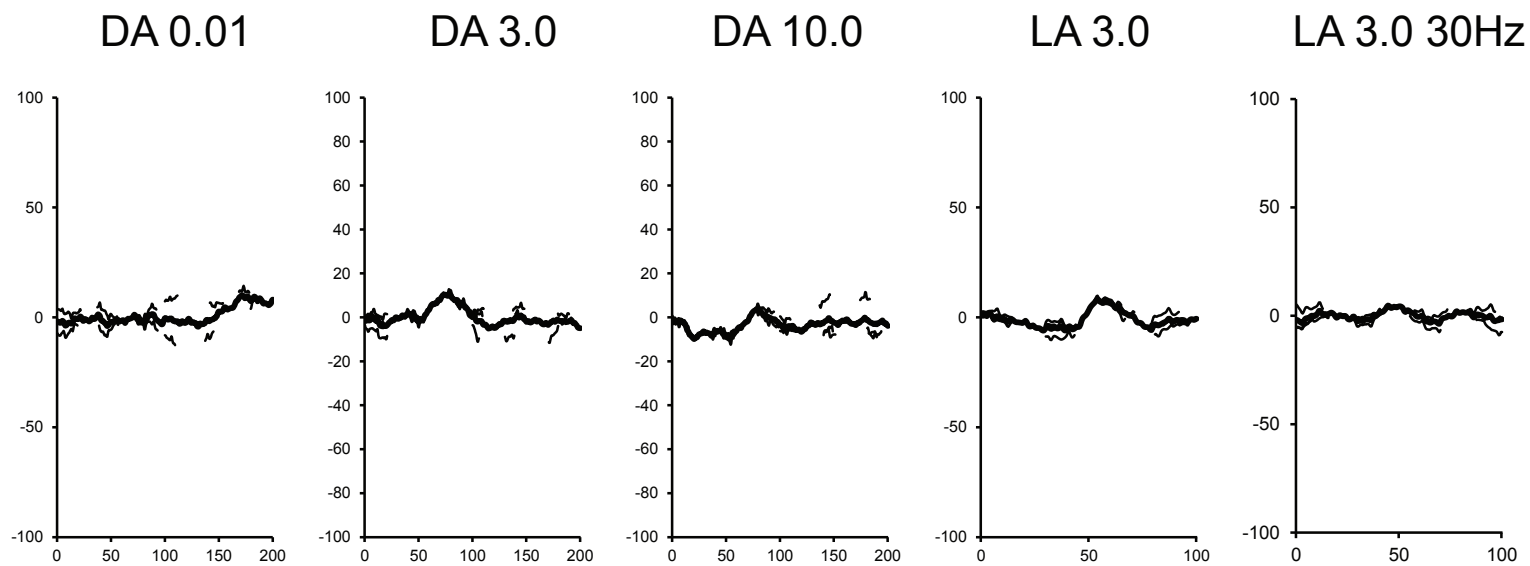

Control

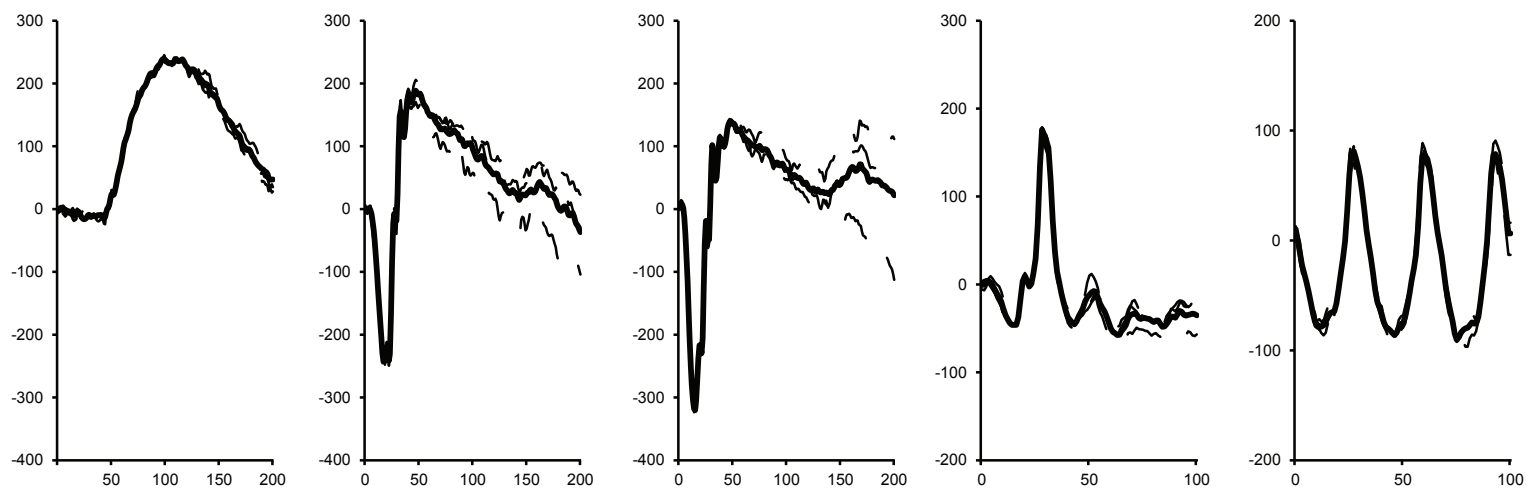

Supplement: Supplementary file 1 [file MGG3-7-na-s001.pdf]

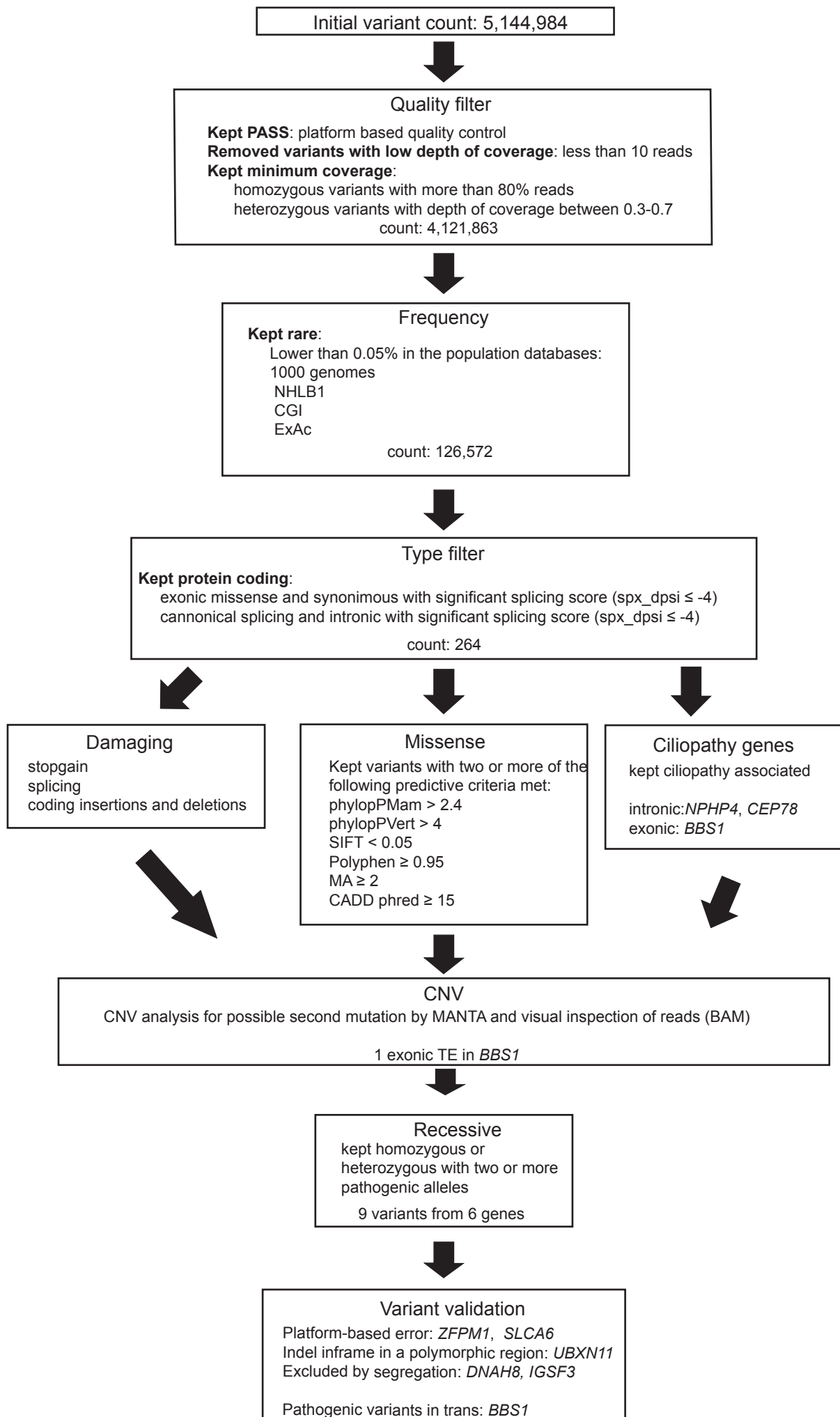

Supplement: Supplementary file 2 [file MGG3-7-na-s002.pdf]
