# Supplementary material for: Retrotransposon insertion as a novel mutational event in Bardet‐Biedl syndrome
Source: Mol Genet Genomic Med. 2018 Nov 28;7(2):e00521. doi: 10.1002/mgg3.521 (PMC6393654; doi:10.1002/mgg3.521)

A

```
wt      1 MAAASSSDSDACGAESNEANSKWLDAHYDPMANIHTFSACLALADLHGDGEYKLVVGDLG   60
mut     1 MAAASSSDSDACGAESNEANSKWLDAHYDPMANIHTFSACLALADLHGDGEYKLVVGDLG

wt     61 PGGQQPRLKVLKGPLVMTESPLPALPAAAATFLMEQHEPRTPALALASGPCVYVYKNLRP  120
mut       PGGQQPRLKVLKGPLVMTESPLPALPAAAATFLMEQHEPRTPALALASGPCVYVYKNLRP

wt    121 YFKFSLPQLPPNPLEQDLWNQAKEDRIDPLTLKEMLESIRETAEEPLSIQSLRFLQLELS  180
mut       YFKFSLPQLPPNPLEQDLWNQAKEDRIDPLTLKEMLESIRETAEEPLSIQSLRFLQLELS

wt    181 EMEAFVNQHKSNSIKRQTVITTMTTLKKNLADEDAVSCLVLGTENKELLVLDPEAFTILA  240
mut       EMEAFVNQHKSNSIKRQTVITTMTTLKKNLADEDAVSCLVLGTENKELLVLDPEAFTILA

wt    241 KMSLPSVPVFLEVSGQFDVEFRLAAACRNGNIYILRRDSKHPKYCIELSAQPVGLIRVHK  300
mut       KMSLPSVPVFLEVSGQFDVEFRLAAACRNGNIYILRRDSKHPKYCIELSAQPVGLIRVHK

wt    301 VLVVGSTQDSLHGFTHKGKKLWTVQMPAAILTMNLLEQHSRGLQAVMAGLANGEVRIYRD  360
mut       VLVVGSTQDSLHGFTHKGKKLWTVQMPAAILTMNLLEQHSRGLQAVMAGLANGEVRIYRD  360

wt    361 KALLNVIHTPDAVTSLCFGRYGREDNTLIMTTRGGGLIIKILKRTAVFVEGGSEVGPPPA  420
mut   361 KALLNVIHTPDAVTSLCFGRYGREDNTLIMTTRGGGLIIKILKRTQCSMVPRLECSGVIS  420

wt    421 QAMKLNVPRKTRLYVDQTLREREAGTAMHRAFQTDLYLLRLRAARAYLQALESSLSPLST  480
mut   421 AHYNLHLPAACLGLPKCRDCSLCPAATPSGK-----------------------------  451

wt    481 TAREPLKLHAVVQGLGPTFKLTLHLQNTSTTRPVLGLLVCFLYNEALYSLPRAFFKVPLL  540
mut       ------------------------------------------------------------

wt    541 VPGLNYPLETFVESLSNKGISDIIKVLVLREGQSAPLLSAHVNMPGSEGLAAA-------  593
mut       ------------------------------------------------------------
```

B

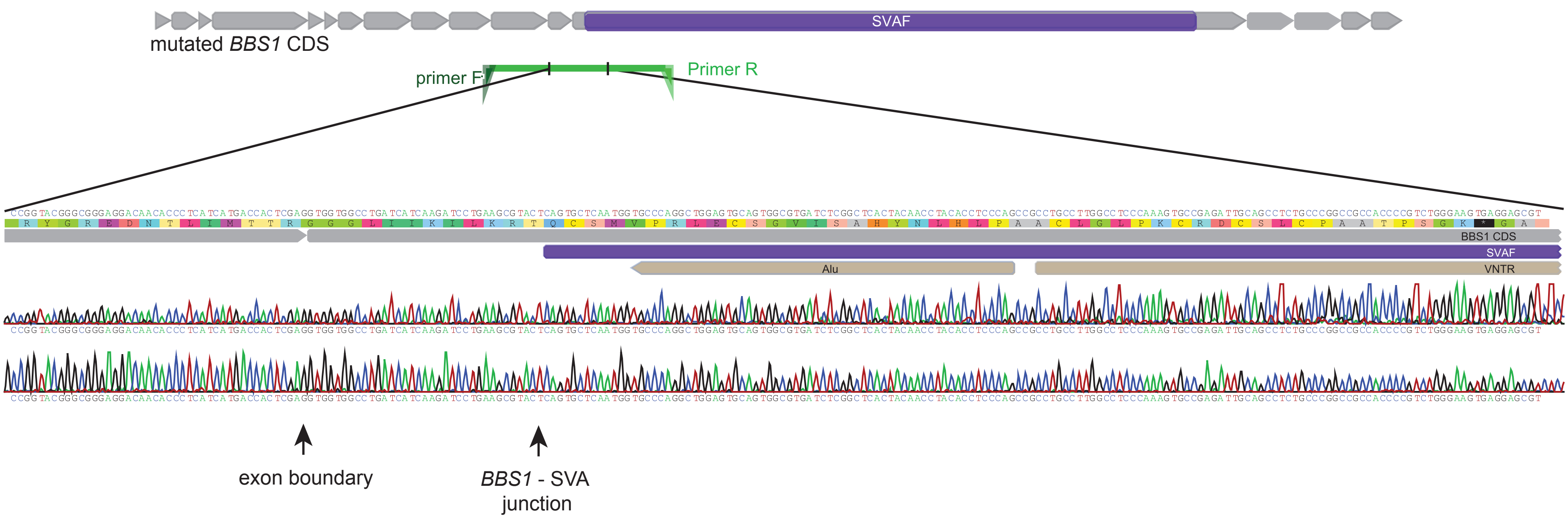

Supplement: Supplementary file 4 [file MGG3-7-na-s004.pdf]
