# Supplementary material for: Retrotransposon insertion as a novel mutational event in Bardet‐Biedl syndrome
Source: Mol Genet Genomic Med. 2018 Nov 28;7(2):e00521. doi: 10.1002/mgg3.521 (PMC6393654; doi:10.1002/mgg3.521)
Supplement: Supplementary file 5 [file MGG3-7-na-s005.docx]

Supp. Table S1. Primer sequences designed to amplify the SVA inserted in BBS1 exon 13 of the proband. Primer ID corresponds to putative map position in Figure S2.

| Primer name | Primer Sequence | Direction |
| --- | --- | --- |
| P1847 | GGTAGAACTGGGGAGGACAA | Forward |
| P5104 | CATCAAGATCCGGAAGCGTACTCAG | Forward |
| P5237 | CCATCTGGGATGTGAGGAGC | Forward |
| P5230 | GGTTGGGGGTAAGGTCACAG | Reverse |
| P5115 | CTTCCGCAGTGTTTGTGTCC | Reverse |
| P5112 | GGACACAAACACTGCGGAAG | Reverse |
| P5113 | TCTGATCCACGTAAAGCCGG | Reverse |
