# Supplementary material for: Retrotransposon insertion as a novel mutational event in Bardet‐Biedl syndrome
Source: Mol Genet Genomic Med. 2018 Nov 28;7(2):e00521. doi: 10.1002/mgg3.521 (PMC6393654; doi:10.1002/mgg3.521)
Supplement: Supplementary file 6 [file MGG3-7-na-s006.docx]

**Methods**

**Next- Generation Sequencing and Variant Analysis**

Pair- end (2 x 100 bp) Whole Exome Sequencing (WES) was performed in the Illumina Hi-Seq 2500 platform after target enrichment of 3µg of genomic DNA using the Agilent SureSelect Human Exome Library V5 kit. Overall mean exon coverage in trio-1 was 87-111X with >20X target base coverage of 96-97%. Overall mean exon coverage in trio-2 was 68-131X with >20X base coverage of 92-97%. Paired-end (2 x 150 bp) Whole Genome Sequencing (WGS) was performed using Illumina Hi-Seq X with mean coverage of 39.4×.

Base calling for was WES and WGS was performed using Illumina HiSeq Analysis Software (HAS; version 2-2.5.55.1311). Reads were mapped to the human b37 reference sequence using bwa-mem v0.7.12 (Li & Durbin, 2010). Duplicate reads were removed using MarkDuplicates from Picard v2.5.0. Local read realignment around indels, base quality score recalibration (BQSR), variant calling with HaplotypeCaller, and variant quality score recalibration (VQSR) were accomplished using GATK v3.7.0 (McKenna et al., 2010).

Resulting variant calls were annotated using a custom pipeline developed at The Centre for Applied Genomics (TCAG) at SickKids based on ANNOVAR (K. Wang, Li, & Hakonarson, 2010). Copy number variants for WGS were called using the read depth method by the programs ERDS v1.1 and CNVnator v0.3.2 using a window size of 500 bp (Abyzov, Urban, Snyder, & Gerstein, 2011; Zhu et al., 2012). Structural variants for WGS were called using Manta v0.29.6 and LUMPY v0.2.13 (Chen et al., 2016; Layer, Chiang, Quinlan, & Hall, 2014). LUMPY calls were genotyped using SVTyper v0.1.2 (Chiang et al., 2015). Variant filtering for WGS was done using the filtering pipeline shown in Supp. Figure S1. The binary alignment map (BAM) generated from bwa-mem from WGS amd WES were used for transposable elements (TE) detection performed by mobile element insertion detection software Mobster v0.2.4.1 with the following parameters: minimum_mapq_anchor=20, use_split=true, minimum_clip_length=15, maximum_clip_length=7, minimum_avg_quality=5, minimum_polyA_length=5, maximum_mismatches_polyA=1, minimum_supporting_reads=3 (Thung et al., 2014). The effect of the predicted TE insertion points on known genes was determined by ANNOVAR (K. Wang et al., 2010).

**TE identification**

We identified two sets of split reads in *BBS1* (NM_024649) exon 13 on the BAM file of the patient WGS by visual inspection in IGV (Robinson et al., 2011). The first set contains reads with poly tail A from position c.1198 that point toward the 3’ direction. The second set contains reads with varied nucleotides starting at position c.1214 that point toward 5’ end (Figure 2A). Both sets of reads and their corresponding mates were collected in order to keep the read pair correspondence and direction. Each set of reads was aligned to exon 13 of *BBS1*, and the consensus sequence of the split region from the second set of split reads (query 127 bp) were used for BLAT analysis at the UCSC portal (<https://genome.ucsc.edu/cgi-bin/hgGateway>) using the GRCh37/hg19 human genome. The output indicated that it has 98.5 % match with over 30 regions on more than 20 different chromosomes. By using the RepeatMasker (<http://www.repeatmasker.org/>), we verified the mapping of all those regions to a short interspread nuclear element (SINE)- variable number tandem repeat (VNTR)-*Alu* type F (SVAF) retrotransposable element (first 127 bp of query had 98.5% match with hg19-chr7: 72697295-72699357 and other regions). SVA is a category of non-Long Terminal Repeat (LTR) retrotransposon, which replicated through target primed reverse transcription (TPRT). Thus, polyadenylation is generally observed at its 3’ end (Ewing, 2015; Hancks & Kazazian, 2010; H. Wang et al., 2005). We identified the polyadenylation in the first set of the ‘split’ reads in our patient (Figure 2A). The TPRT also creates a target site duplication (TSD) of 7-20 bp on the BBS-SVA junction, which was also identified in our patient reads as a17 bp from positions NM_024649:c.1198-1214 of *BBS1* (Figure 2A,C).

A predicted map of the mutation event (Supplemental Figure 2) was constructed using a close SVAF match to our target from the UCSC database as a reference (hg19_rmsk_SVA_F range= chr7 :72697295-72699357). We mapped the exported next generation split reads from the patient and their corresponding mates onto our SVAF reference using Geneious 8.1.8 (Kearse et al., 2012). The mapped reads validated the insertion point of the SVA, TSD, and the overall map (Figure 2C, Supp. Material 2). Moreover, this map allowed us to design primers for Sanger sequencing to extend the known sequence of the inserted TE in our patien. This was important because next generation sequencing data from inserts within retroelements are not reliable because of their repetitive nature (Table S1, Supp. Figure S2). PCR amplification of the SVA from the total DNA of the proband, father and mother was performed using Amplitaq Gold 360 master mix (*ThermoFisher*) in 25 µL reaction with 250 nM of each primer (two amplicons from Figure S2B), and 3 µL of the provided GC enhancer. PCR conditions: 10 minutes of initial denaturation at 95^o^C; touch down for 10 cycles of 30 seconds denaturation at 95^o^C, 30 seconds of annealing from 71-61^o^C (reducing 1^o^C/ cycle), 3 minutes of extension for the large template and 30 seconds for the short one at 72 ^o^C; another 30 cycles at with same overall conditions, but with constant annealing temperature at 61 ^o^C; final extension of 7 minutes at 72 ^o^C. The SVA segments were obtained from the father and the proband’s DNA due to the lack of the TE in the mother. PCR products were purified with QIAEXII gel extraction kit (*Qiagen*) according to manufacture’s instructions. Sanger sequencing was performed at TCAG using the same amplification primers, and additional internal primer for the longer template with 1µL GC enhancer to avoid secondary structure on the highly GC –rich portions of the SVA (Figure S2B, Table S1). The large purified PCR product was cloned using CloneJet kit (*ThermoFisher*) in TOP10 bacterial strain. The plasmid with the SVA sequence was sequenced, however the segment was truncated due to secondary structure of the GC-rich tandem repeats. The insert in the plasmid had 100% match with the directed sequenced segments. The final insert and adjacent areas sequence was inferred with the consensus of all the patient reads (GenBank MH395756). The inserted SVA was ~95% similar to multiple SVA type F mapped in the human genome (UCSC with RepeatMasker track). Using Dfam database (<http://www.dfam.org/>) , some parts of our SVA insertion had higher similarity with SVA types B and D besides F. SINE, VNRT region and Alu were annotated according previous SVA characterization studies (Hancks & Kazazian, 2010; Hancks, Mandal, Cheung, & Kazazian, 2012; Savage, Bubb, Breen, & Quinn, 2013).

**RNA expression**

Total RNA was extracted from patient derived the lymphoblast cell lines (generated at the Biobank of the TCAG – Sickkids) using Rneasy Plus Mini Kit (*Qiagen*) with lysis buffer. A cDNA was synthesized from the RNA using Superscript IV enzyme kit (*Thermofisher*). RT-PCR reaction was performed using a primer forward (CACAAGGGGAAGAAGCTGTG) designed at the boundary between exons 10 and 11, and the primer reverse (CCTTACTTCCTAGATGGGATGGC) was designed within the SVA element downstream to the stop codon (Figure S4). RT-PCR was performed in 12.5 uL reaction with Platinum Superfi DNA Polymerase (*Thermofisher*) using the GC-rich enhancer (*Thermofisher*). The RT-PCR product matched the expected size (~529 bp) and was Sanger sequenced, validating the expression of the allele with the SVA insertion, and its predicted effect (Figure S4).

References

Abyzov, A., Urban, A. E., Snyder, M., & Gerstein, M. (2011). CNVnator: an approach to discover, genotype, and characterize typical and atypical CNVs from family and population genome sequencing. *Genome Res, 21*(6), 974-984. doi:10.1101/gr.114876.110

Chen, X., Schulz-Trieglaff, O., Shaw, R., Barnes, B., Schlesinger, F., Kallberg, M., . . . Saunders, C. T. (2016). Manta: rapid detection of structural variants and indels for germline and cancer sequencing applications. *Bioinformatics, 32*(8), 1220-1222. doi:10.1093/bioinformatics/btv710

Chiang, C., Layer, R. M., Faust, G. G., Lindberg, M. R., Rose, D. B., Garrison, E. P., . . . Hall, I. M. (2015). SpeedSeq: ultra-fast personal genome analysis and interpretation. *Nat Methods, 12*(10), 966-968. doi:10.1038/nmeth.3505

Ewing, A. D. (2015). Transposable element detection from whole genome sequence data. *Mob DNA, 6*, 24. doi:10.1186/s13100-015-0055-3

Hancks, D. C., & Kazazian, H. H., Jr. (2010). SVA retrotransposons: Evolution and genetic instability. *Semin Cancer Biol, 20*(4), 234-245. doi:10.1016/j.semcancer.2010.04.001

Hancks, D. C., Mandal, P. K., Cheung, L. E., & Kazazian, H. H., Jr. (2012). The minimal active human SVA retrotransposon requires only the 5'-hexamer and Alu-like domains. *Mol Cell Biol, 32*(22), 4718-4726. doi:10.1128/MCB.00860-12

Kearse, M., Moir, R., Wilson, A., Stones-Havas, S., Cheung, M., Sturrock, S., . . . Drummond, A. (2012). Geneious Basic: an integrated and extendable desktop software platform for the organization and analysis of sequence data. *Bioinformatics, 28*(12), 1647-1649. doi:10.1093/bioinformatics/bts199

Layer, R. M., Chiang, C., Quinlan, A. R., & Hall, I. M. (2014). LUMPY: a probabilistic framework for structural variant discovery. *Genome Biol, 15*(6), R84. doi:10.1186/gb-2014-15-6-r84

Li, H., & Durbin, R. (2010). Fast and accurate long-read alignment with Burrows-Wheeler transform. *Bioinformatics, 26*(5), 589-595. doi:10.1093/bioinformatics/btp698

McKenna, A., Hanna, M., Banks, E., Sivachenko, A., Cibulskis, K., Kernytsky, A., . . . DePristo, M. A. (2010). The Genome Analysis Toolkit: a MapReduce framework for analyzing next-generation DNA sequencing data. *Genome Res, 20*(9), 1297-1303. doi:10.1101/gr.107524.110

Robinson, J. T., Thorvaldsdottir, H., Winckler, W., Guttman, M., Lander, E. S., Getz, G., & Mesirov, J. P. (2011). Integrative genomics viewer. *Nat Biotechnol, 29*(1), 24-26. doi:10.1038/nbt.1754

Savage, A. L., Bubb, V. J., Breen, G., & Quinn, J. P. (2013). Characterisation of the potential function of SVA retrotransposons to modulate gene expression patterns. *BMC Evol Biol, 13*, 101. doi:10.1186/1471-2148-13-101

Wang, H., Xing, J., Grover, D., Hedges, D. J., Han, K., Walker, J. A., & Batzer, M. A. (2005). SVA elements: a hominid-specific retroposon family. *J Mol Biol, 354*(4), 994-1007. doi:10.1016/j.jmb.2005.09.085

Wang, K., Li, M., & Hakonarson, H. (2010). ANNOVAR: functional annotation of genetic variants from high-throughput sequencing data. *Nucleic Acids Res, 38*(16), e164. doi:10.1093/nar/gkq603

Zhu, M. F., Need, A. C., Han, Y. J., Ge, D. L., Maia, J. M., Zhu, Q. Q., . . . Goldstein, D. B. (2012). Using ERDS to Infer Copy-Number Variants in High-Coverage Genomes. *American Journal of Human Genetics, 91*(3), 408-421. doi:10.1016/j.ajhg.2012.07.004
