# Supplementary material for: Retrotransposon insertion as a novel mutational event in Bardet‐Biedl syndrome
Source: Mol Genet Genomic Med. 2018 Nov 28;7(2):e00521. doi: 10.1002/mgg3.521 (PMC6393654; doi:10.1002/mgg3.521)
Supplement: Supplementary file 3 [file MGG3-7-na-s003.pdf]

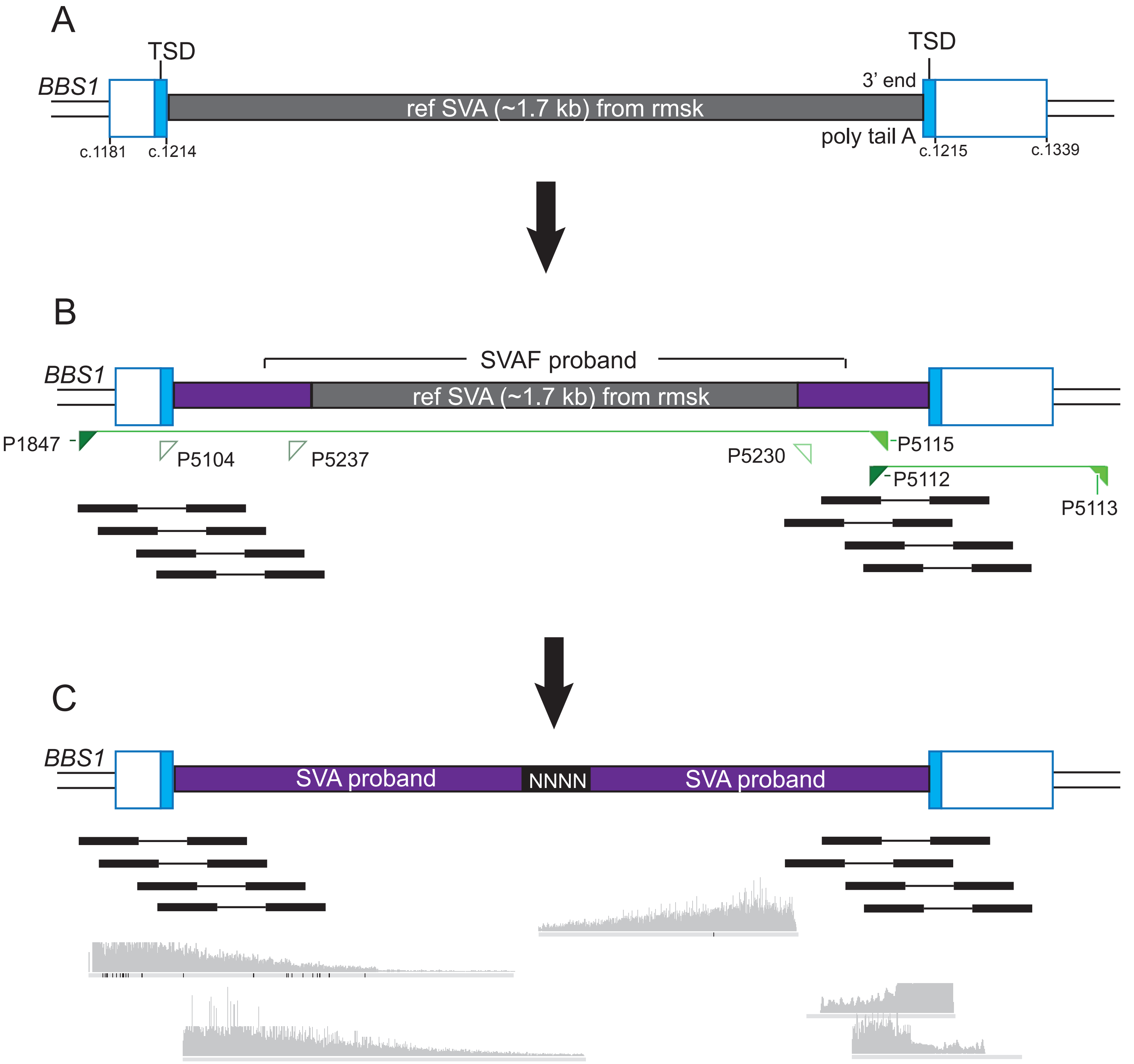
A
TSD
BBS1
3' end
TSD
ref SVA (~1.7 kb) from rmsk
poly tail A
c.1181
c.1214
c.1215
c.1339
B
SVAF proband
BBS1
ref SVA (~1.7 kb) from rmsk
P1847
P5104
P5237
P5230
P5115
P5112
P5113
C
BBS1
SVA proband
NNNN
SVA proband
